# A 20-Year Post-traumatic Presentation of a Horizontal Root Fracture Accompanied by Bone Resorption and Sinus Tract: A Case Report

**DOI:** 10.7759/cureus.100815

**Published:** 2026-01-05

**Authors:** Pedram Hosseinzadehfard, Paulina Bernote, Greta Lodiene

**Affiliations:** 1 Department of Dental and Oral Pathology, Lithuanian University of Health Sciences, Kaunas, LTU; 2 Faculty of Odontology, Lithuanian University of Health Sciences, Kaunas, LTU

**Keywords:** apical surgery, dental trauma, horizontal root fracture, late-pulp necrosis, microsurgical endodontics

## Abstract

Horizontal root fractures present unique diagnostic and treatment challenges, particularly in cases with delayed presentation. This report describes the management of a 20‑year‑old untreated horizontal root fracture of the maxillary left central incisor, complicated by the presence of a persistent sinus tract. A 56‑year‑old male patient was referred due to mild anterior maxillary pain accompanied by purulent discharge. Clinical examination revealed Grade II mobility and tenderness upon percussion of tooth 21. Fistulogram traced the sinus tract to a displaced apical fragment of tooth 21. Endodontic treatment was conducted on the coronal fragment under magnification, including canal shaping, calcium hydroxide dressing, and final obturation with Biodentine (Septodont, Saint-Maur-des-Fosses, France). The persistence of the sinus tract during follow‑up indicated necrosis of the apical fragment. Surgical removal was performed using a papilla‑based flap technique under a dental operating microscope, ensuring the preservation of the surrounding periodontal and osseous structures. Healing progressed uneventfully, with complete resolution of the sinus tract and stability of the coronal fragment confirmed at 3‑, 6‑, and 12‑month post-treatment.

This case highlights the potential for late‑onset pulp necrosis occurring decades after trauma in horizontal root fractures and emphasizes the importance of individualized management strategies. Selective endodontic treatment of the coronal fragment, in conjunction with the surgical removal of a necrotic apical fragment, may represent a conservative and effective approach that preserves tooth function and obviates complete extraction in chronic presentations with persistent pathology. Early diagnosis and periodic monitoring are critical for preventing such long‑term complications.

## Introduction

Healing following a root fracture initiates locally, encompassing the pulp and periodontal tissues. In the absence of infection, a fracture may undergo healing through the formation of hard tissue or via the interposition of connective tissue between the fractured fragments [1]. 

In most cases, the pulp survives the trauma, remaining healthy and preserving normal functionality [2]. Pulp necrosis is relatively uncommon in the apical fragment; however, it occurs with greater prevalence in the coronal fragment. Endodontic treatment of the coronal fragment is indicated when the fracture line fails to demonstrate signs of healing [3]. Nevertheless, delayed pulp necrosis and infection may manifest many years subsequent to the initial injury. This phenomenon suggests that the pulp remained functional until a subsequent event, such as periodontal disease, dentin-involving infraction, or additional dental trauma - facilitated the ingress of bacteria into the root canal system [4].

When necrosis affects pulp tissue from both coronal and apical fragments, treatment becomes significantly more complex and presents additional clinical challenges. Endodontic intervention across the fracture line may compromise the integrity of the surrounding periodontal tissues. Alternatively, surgical removal of the apical fragment may be considered, contingent upon the coronal fragment maintaining adequate support and periodontal attachment [5].

Several factors can influence the long-term prognosis of a tooth subjected to a transverse root fracture, including the location of the fracture line, patient age, distance between the fractured segments, exposure of the fracture site to the oral cavity, and any pre-existing conservative, endodontic, or periodontal conditions [6]. Delayed treatment may result in significant long-term consequences. Teeth that experience trauma and receive late or no treatment are more susceptible to developing pulp canal obliteration, pulp necrosis, root resorption, and loss of marginal bone support [7].

This case report details the management of a horizontal root fracture associated with a sinus tract in a 56‑year‑old patient, presenting 20 years after the original traumatic incident.

## Case presentation

The present case report followed the guidelines of Preferred Reporting Items for Case Reports in Endodontics 2020 (PRICE) [8]. Written informed consent was obtained from the patient for reporting this case. A 56-year-old male patient was referred to the Department of Dental and Oral Pathology of the Lithuanian University of Health Sciences, Kaunas, Lithuania, complaining of mild pain in the anterior maxilla. The patient’s medical history revealed facial trauma approximately 20 years prior, which resulted in a horizontal root fracture of tooth 21. No treatment was initiated at that time, and due to the absence of symptoms in subsequent years, the patient did not seek further follow-up.

**Figure 1:**
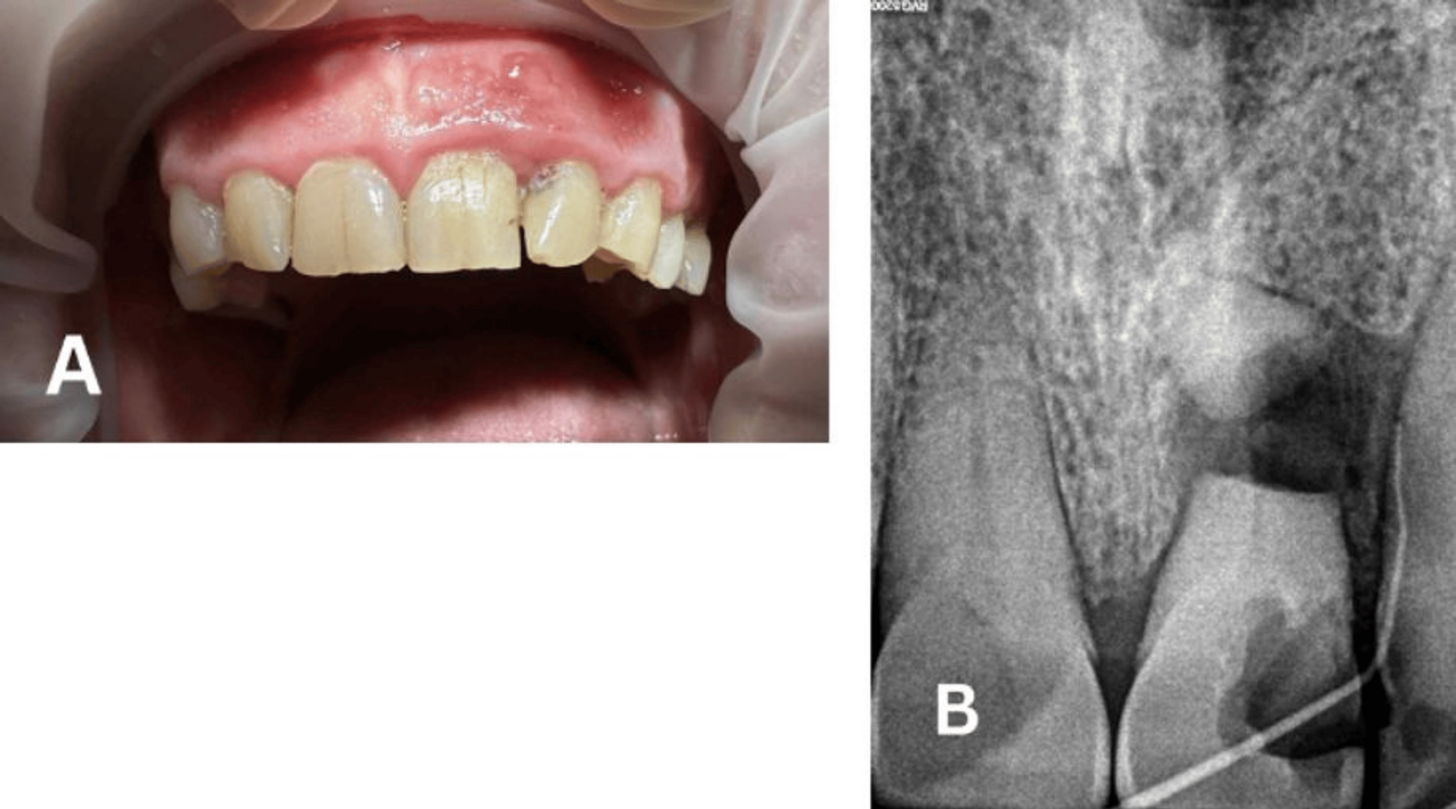
(A) Initial Clinical Picture and (B) Initial Radiograph (Fistulogram)

Initial clinical examination revealed Grade II mobility according to the Miller classification, in addition to tenderness to percussion and a persistent sinus tract exhibiting purulent exudate (Figure 1A). The initial radiographic examination (fistulogram) demonstrated a horizontal root fracture with segment displacement, and the sinus tract was traced using gutta-percha #20 (Figure 1B).

**Figure 2:**
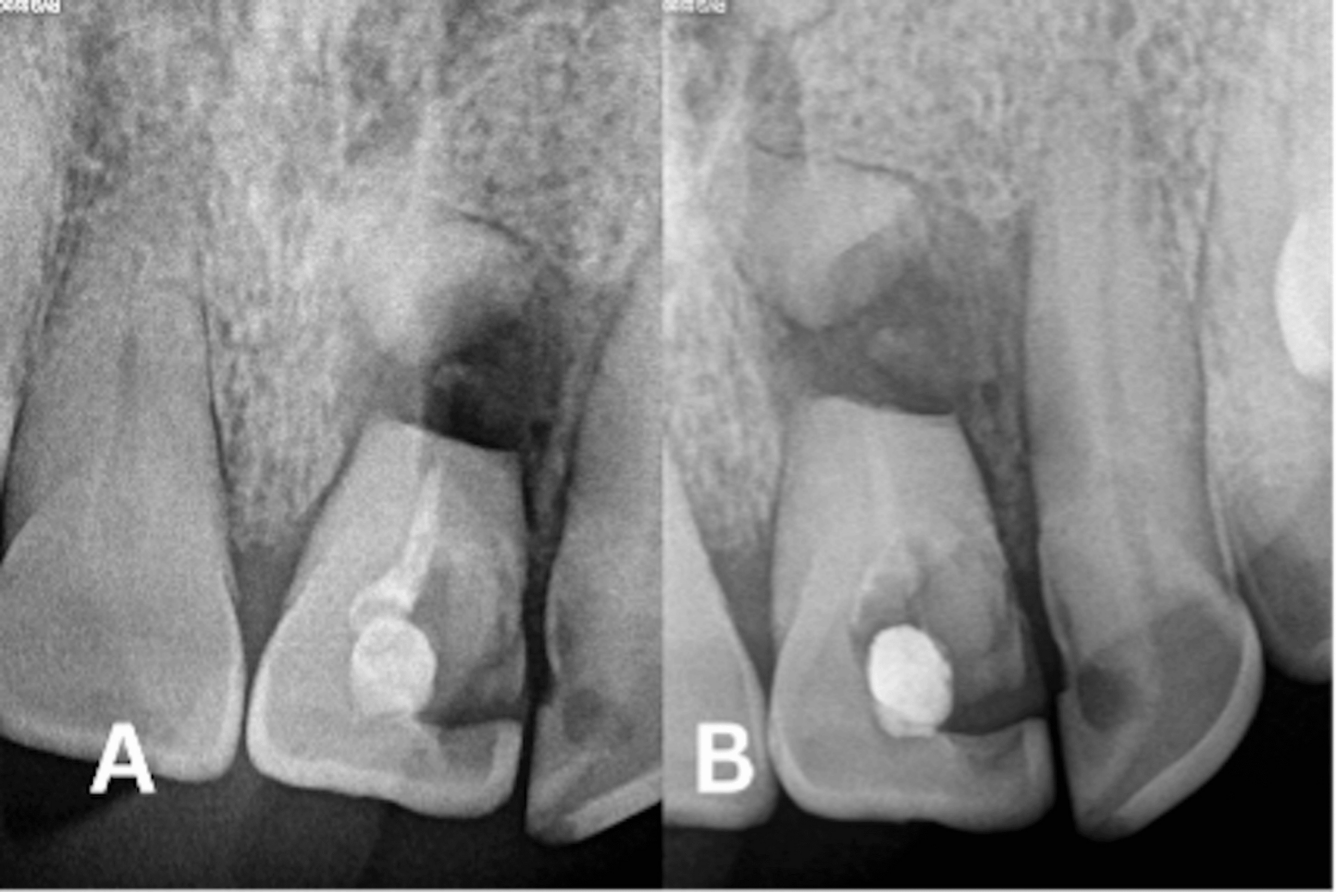
(A) Coronal Part Filled With (CaOH)2 and (B) Coronal Canal Obturated With Biodentine

During the initial visit, the tooth was isolated using a rubber dam system, and endodontic treatment was initiated. The endodontic access cavity was prepared under a surgical microscope (Leica Microsystem GmbH, Wetzlar, Germany) with a high-speed diamond bur under water coolant. A K-file size #10 was used to explore the canal, with the coronal portion of the working length determined to be 20 mm from the incisal edge to the fracture. The coronal segment of the root canal was shaped using hand instruments in a crown-down technique up to K-file #60. Subsequently, a calcium hydroxide medication (Ultracal, Ultradent Products Inc., South Jordan, UT, USA) was applied to the coronal fragment, and a follow-up appointment was scheduled two weeks later (Figure 2A).

During the second dental appointment, a persistent sinus tract was observed, which confirmed the diagnosis of pulp necrosis of the apical fragment. The tooth was isolated using a rubber dam, and the access cavity was re-entered. The calcium hydroxide dressing was meticulously removed, with irrigation of the canal performed following each instrument change. Subsequently, the root canal was irrigated with 5 mL of 0.5% sodium hypochlorite (NaOCl), and a final rinse was carried out using sterile water. Following this, the coronal fragment was thoroughly irrigated with 5 mL of 17% ethylenediaminetetraacetic acid (EDTA) solution, irrigated with sterile water, dried, and then obturated with Biodentine (Septodont, Saint-Maur-des-Fosses, France) (Figure 2B). The endodontic access cavity was sealed with glass ionomer cement (Riva, Victoria, Australia).

At the third appointment, the patient was admitted for the surgical extraction of the apical fragment. Local anesthesia, comprising 2% lidocaine with 1:100,000 epinephrine (Xylestesin-A 2%, 3M ESPE, Seefeld, Germany), was administered via a nasopalatine nerve block with concomitant (labial) buccal infiltration at the mucolabial fold. Additionally, one cartridge was administered as a supplemental buccal infiltration.

**Figure 3:**
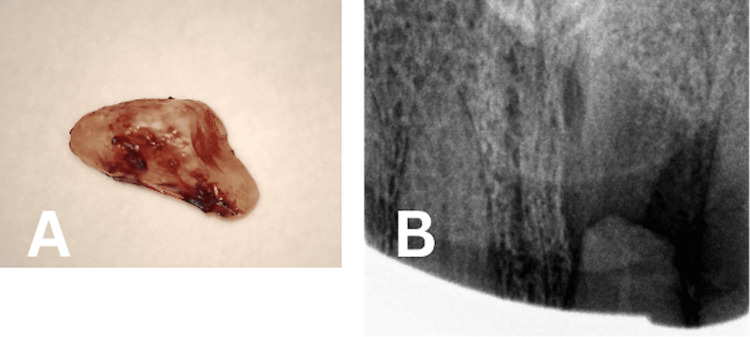
(A) Removed Apical Fragment and (B) Postoperative Radiograph After Apical Fragment Removal

A full mucoperiosteal flap utilizing a papilla-based incision technique was selected to preserve the interdental papilla, maintain optimal esthetics in the anterior region, and minimize the risk of postoperative recession compared to conventional flap designs. Gingiva and mucosa were incised using a 15C blade. A partial-thickness flap was elevated at the sulcus and base of the papilla, followed by the elevation of a full mucoperiosteal flap extending to the mucosa. A flap with one vertical incision was created, extending from the distal surface of tooth 11 to the distal surface of tooth 22. Granulation tissue was subsequently excised, and the apical fragment was extracted (Figure 3A).

**Figure 4:**
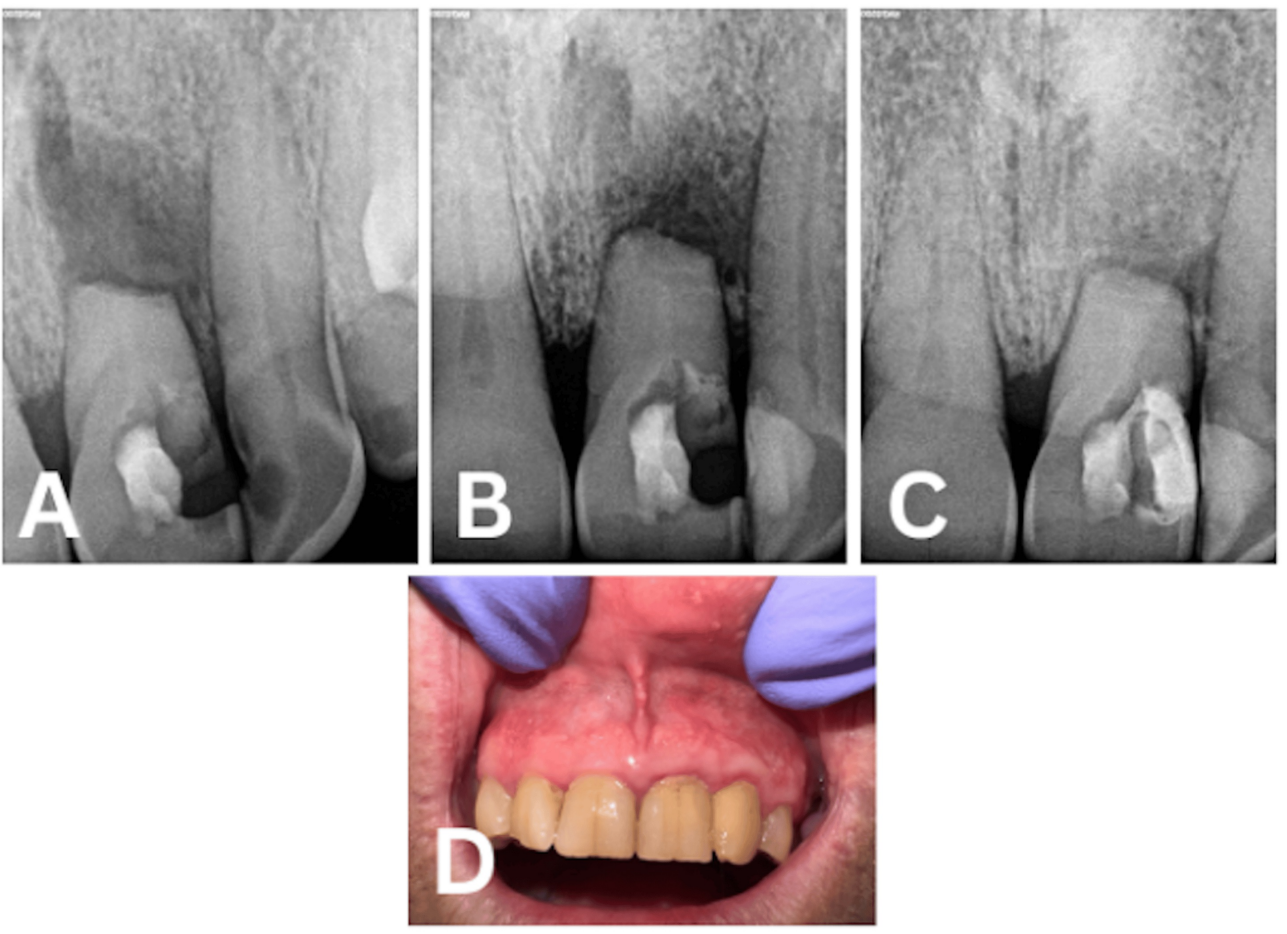
Follow-Ups (A) After Three Months, (B) After Six Months, (C) After One Year, and (D) Clinical Picture After One Year

Subsequently, the surgical site was meticulously irrigated with saline, and the flap was fixated and sutured using a 5-0 suture. The entire endodontic surgical procedure was performed under a dental operating microscope (Leica Microsystem GmbH, Wetzlar, Germany). A postoperative two-dimensional radiograph was obtained (Figure 3B). Postoperative instructions concerning oral hygiene practices for the surgical site and dietary intake were provided. Non-steroidal anti-inflammatory drugs (NSAIDs) were recommended for postoperative pain management if required. Follow-up appointments were scheduled for suture removal at two weeks as well as at three months, six months, and one year postoperatively (Figure 4). At the two-week visit, complete resolution of the sinus tract was observed. Mobility of the coronal segment progressively decreased at the three- and six-month evaluations. Radiographic assessment at six months and one year demonstrated evidence of bone healing, indicating a favorable clinical and radiographic outcome.

## Discussion

Although pulp necrosis may occur following horizontal root fractures, immediate endodontic intervention is generally not recommended. Instead, periodic clinical and radiographic monitoring is advised in the absence of clinical or pathological signs. Frequent follow-up visits are necessary to assess the need for endodontic treatment [9]. In the present case, the existence of a persistent sinus tract indicated the presence of infection and necrosis, thereby necessitating endodontic treatment of the coronal fragment 20 years post-trauma.

In cases of pulp necrosis associated with horizontal root fractures, it is frequently noted that the apical fragment maintains vitality; consequently, endodontic therapy is typically limited to the coronal segment [10]. However, case reports over the last years have noted that even apical third fractures can demonstrate delayed pathological changes when long intervals occur between trauma and treatment, although these reports are relatively uncommon compared with middle third fractures [11]. In the current case study, despite the execution of adequate chemomechanical preparation, the application of intracanal medication, and the obturation of the coronal portion, the persistence of a sinus tract suggested the possible existence of necrosis or infection within the apical fragment. Therefore, a surgical intervention for the removal of the apical segment was deemed necessary. The extraction of the apical fragment was conducted under magnification to facilitate thorough debridement while preserving the surrounding bone and soft tissues. Recent literature supports this approach, demonstrating that apical fragment removal can promote healing when chronic infection persists, while maintaining periodontal support and function of the coronal fragment [12].

Although conservative management and retention of both fragments are generally preferred, the extraction of the apical fragment may be warranted in instances of persistent pathology, particularly when the coronal segment remains stable and functional [13]. In the current case, this approach facilitated the resolution of the sinus tract while preserving the coronal portion of the tooth, thereby illustrating that the selective removal of the necrotic apical fragment can serve as an effective and conservative alternative to complete extraction in chronic cases of root-fractured teeth exhibiting persistent periapical pathology. Furthermore, the long-standing periapical lesion in this case may suggest that the inflammatory process has extended to the apical fragment, potentially leading to its loss of vitality [14].

In similar cases detected shortly after trauma, prompt diagnosis, appropriate stabilization, and close monitoring of pulp vitality are essential to guide timely intervention and avoid progression of pathology. Early, targeted management can prevent the need for complete extraction and help preserve the remaining periodontal support, thereby maintaining both function and esthetics.

## Conclusions

Late pulp necrosis can manifest decades following a horizontal root fracture, underscoring the importance of long-term follow-up for traumatized teeth. Targeted removal of the infection source, in conjunction with endodontic treatment of the coronal segment, presents a conservative and functional alternative to complete extraction.
